# New Nanofibers Based on Protein By-Products with Bioactive Potential for Tissue Engineering

**DOI:** 10.3390/ma13143149

**Published:** 2020-07-15

**Authors:** Maria Râpă, Carmen Gaidău, Laura Mihaela Stefan, Ecaterina Matei, Mihaela Niculescu, Mariana Daniela Berechet, Maria Stanca, Cristina Tablet, Mădălina Tudorache, Raluca Gavrilă, Cristian Predescu, Ruxandra Vidu

**Affiliations:** 1 Faculty of Material Sciences and Engineering, Politehnica University of Bucharest, 313 Spl. Independentei, 060042 Bucharest, Romania; rapa_m2002@yahoo.com (M.R.); ecaterinamatei@gmail.com (E.M.); cpredescu56@yahoo.com (C.P.); r_vidu@yahoo.com (R.V.); 2 National Research and Development Institute for Textiles and Leather- Division Leather and Footwear Research Institute, 031215 Bucharest, Romania; mihaela.niculescu@icpi.ro (M.N.); marianadanielaberechet@yahoo.co.uk (M.D.B.); maria.stanca@icpi.ro (M.S.); 3 National Institute of Research and Development for Biological Sciences, 296 Splaiul Independenţei, 060031 Bucharest, Romania; lauramihaelastefan@yahoo.com; 4 Department of Physical Chemistry, University of Bucharest, 4–12 Blvd. Regina Elisabeta, 030018 Bucharest, Romania; cristinatablet@yahoo.com (C.T.); madalina.sandulescu@g.unibuc.ro (M.T.); 5 Faculty of Pharmacy, Titu Maiorescu University, Gh. Sincai Bd. 16, 040317 Bucharest, Romania; 6 Nano-scale Structuring and Characterization Laboratory, National Institute for R&D in Microtechnologies, 126A Erou Iancu Nicolae Street, R-077190 Voluntari, Romania; raluca.gavrila@imt.ro; 7 Department of Electrical and Computer Engineering, University of California Davis, One Shields Avenue, Davis, CA 95616, USA

**Keywords:** collagen hydrolysate, rabbit collagen glue, keratin hydrolysate, electrospinning, triple helix, in vitro cytotoxicity

## Abstract

Concentrated collagen hydrolysate (HC10CC), rabbit collagen glue (RCG), and keratin hydrolysate (KH) were investigated in terms of their extraction from mammalian by-products and processing by electrospinning. The electrospun nanofibers were characterized by scanning electron microscopy coupled with the energy dispersive X-ray spectroscopy (SEM/EDS), attenuated total reflectance Fourier transform infrared spectroscopy (ATR-FTIR), differential scanning calorimetry (DSC), and indentation tests. The cytotoxicity of the electrospun nanofibers was conducted on L929 fibroblast cells using MTT and LDH assays and cell morphology observations. The electrospun RCG and KH nanofibers morphology showed an average size of nanofibers ranging between 44 and 410 nm, while the electrospun HC10CC nanofibers exhibited higher sizes. The ATR-FTIR spectra performed both on extracted proteins and electrospun nanofibers showed that the triple helix structure of collagen is partially preserved. The results were in agreement with the circular dichroism analysis for protein extracts. Furthermore, the viscoelastic properties of electrospun KH nanofibers were superior to those of electrospun RCG nanofibers. Based on both in vitro quantitative and qualitative analysis, the electrospun nanofibers were not cytotoxic, inducing a healthy cellular response. The results of new electrospun protein-based nanofibers may be useful for further research on bioactive properties of these nanofibers for tissue engineering.

## 1. Introduction

The research on the biocompatible natural polymers processing by electrospinning for biomedical applications has increased in recent years [1,2]. Nanofibers with different thermoplastic polymers, biomaterials, or active compounds (dyes, drugs, light-sensitive or conductive organics, and piezoelectric materials) [3] can be obtained by electrospinning. The main advantage of electrospun fibers is the greater surface area to volume ratios of 1–3 orders of magnitude larger than thin films made of the same material [4], which allows increased contact with skin, sensing analyte, and pollutant substance, or for other applications. Nanofibers based on ethylene-co-vinyl alcohol (EVOH) [5], xanthan polysaccharide [6], silk fibroin [7], chitosan/poly(ethylene oxide) [8], poly(ethylene oxide) (PEO), and poly(vinyl alcohol) (PVA) [9] have been achieved by electrospinning. Although synthetic polymers show good rheological and mechanical properties, their biocompatibility has some limitations and can produce inflammation [10].

Collagen and keratin are the most valuable natural biomaterials due to their chemical versatility and biological performance. They mimic the extracellular matrices (ECM) of tissues and organs, which consist in a complex composite of fibrous proteins such as collagen and fibronectin, glycoproteins, proteoglycans, and soluble proteins (growth factors, bioactive molecules that support cell adhesion and proliferation). In order to be designed as extracellular matrix of tissues, the mechanical rheology and the ability of protein to bind with other biopolymers need to be proved. Collagen is a natural biopolymer, the most abundant protein in mammals, which is widely used in bone restoration engineering [11], processing of controlled release of drug systems [12], as well as in food [13] and cosmetics [14]. Collagen is an expensive material obtained from bovine and swine hides, as well as fish skins [15,16] and usually used in its native state. Collagen hydrolysate obtained by the enzymatic extraction of bovine tendons was successfully used to increase the biocompatibility and degradability of poly(lactic acid) (PLA) [17,18,19]. The main disadvantage of these PLA biocomposites is the poor compatibility between the collagen hydrolysate and synthetic polymeric matrix. It is already known that the collagen extracted from rabbit skin in water media has the best gluing properties [20], probably due to the different structure of C-terminal region of rabbit collagen chains, which are longer by two amino acids, i.e., alanine and arginine [21]. The special gluing properties of rabbit collagen glue were also attributed to the preservation of subunit components like ß (dimer of *ɑ*-chain) and *ϒ* (trimer of *ɑ*-chain) in gelatin state [22]. Keratin is another structural protein available in large quantities in epithelial cells, feathers, wools, hooves, horn, hair, and claws, having interesting properties regarding the biocompatibility and absorptive affinity for heavy metal ions and volatile organic compounds [23]. Keratin is an ideal material for wound healing due to the amino acid sequence that induces cell adhesion, initiates mitogenic and chemotactic activity, and mediates changes in gene expression, which stimulate wound regeneration [24].

The most difficult steps in the electrospinning process are related to the dispersion of proteins molecules in proper solvents or polymer solutions so that the triple helix structure is preserved [25]. Low concentration of native collagen (1%) and expensive, potential toxic denaturing solvents, such as 1,1,1,3,3,3-hexafluoro-2-propanol (HFIP) and 2,2,2-trifluoroethanol (TFE), for preparation of electrospun collagen scaffold or functional biomaterials have been reported in the literature [23,26,27,28,29]. Beside their corrosive nature, common organic solvents used in collagen dispersion, severely seriously damage its helical structure [8,30,31]. When diluted acetic acid in mixture with ethanol is used, the polyproline type II helices fraction increases [31].

The novelty of this research includes the production of nanofibers from low-molecular weight collagen and keratin hydrolysates with a high content of proteins (13.6% *w*/*v*, 25% *w*/*v*, and 22% *w*/*v* for collagen hydrolysate, rabbit collagen glue, and keratin hydrolysate, respectively) by electrospinning. The objectives of this study were (1) to extract and characterize the collagen hydrolysate (HC10), collagen glue (RCG), and keratin hydrolysate (KH) from bovine tanned leather by-products, pickled rabbit skin, and sheepskin wool, respectively; (2) to characterize the protein-based nanofibers obtained by electrospinning for potential biomedical field application; and (3) to demonstrate that the structure of protein-based nanofibers still preserves the secondary collagen structure without being totally affected by the extraction and electrospinning processing conditions. The nanofibers’ surface and mechanical properties have been confirmed using scanning electron microscopy coupled with the energy-dispersive spectroscopy (SEM-EDS) technique, attenuated total reflectance Fourier transform infrared spectroscopy (ATR FT-IR), differential scanning calorimetry (DSC), and indentation tests (Nanoindenter XP). The biocompatibility (in vitro cytotoxicity) of protein-based nanofibers prepared by electrospinning was also evaluated highlighting the perspective of using protein-based nanofibers in tissue engineering.

## 2. Materials and Methods

### 2.1. Materials

Chitosan from crab shells, which is highly viscous (Sigma) in the form of crystals, is characterized by structural viscosity 1267 MPaxs and sulfated ash 0.2%. Poly(ethylene oxide) (PEO), M_w_ 100,000 g mol^−1^, powder (Alpha Aesar, Kandel, Germany) with a melting point of 65 °C and a density of 1.4539 g/cm^3^ was used. PEO shows good biocompatibility and low toxicity and it is soluble in toluene, tetrahydrofuran, hot water, dimethylformamide, and chloroform, which are solvents generally used in the electrospinning process of biomaterials [32]. Glacial acetic acid (Alfa Aesar, Kandel, Germany) with density of 1.05 g/cm³ and distilled water were used as solvents.

### 2.2. Preparation of HC10CC, RCG and KH Extracts

Collagen (HC10) was extracted from bovine tanned leather by-products by chemical enzymatic hydrolysis in alkaline condition using calcium hydroxide (Chimopar S.A., Bucuresti, Romania) at 80 °C and Alcalase 2.4 L (Novozymes) at 60 °C [33]. HC10CC was concentrated up to ~60% (*w*/*w*), at 60 °C by using the Hei-VAP Rotary Evaporator (Schwabach, Germany), at which the collagen hydrolysate viscosity increased enough for developing spinnable properties. Intermediary processes for total separation of chromium traces were done by successive filtrations through 0.45 μm pore size media and decantation after alkaline and enzymatic hydrolysis stages [34]. The rabbit collagen glue (RCG) was prepared from pickled rabbit skin by boiling of the picked skin, preliminarily crushed in water bath at a temperature of 90 °C, for four hours. Keratin hydrolysate (KH) was prepared by solubilization of sheepskin wool preliminarily degreased with 8% NaOH rotulis (Lach-Nersro) at 80 °C, for 4 h, followed by filtration [35]. The yields for HC10, RCG, and KH extracts were 80%, 60%, and 40%, respectively.

### 2.3. Electrospinning of HC10CC, RCG and KH Extracts

Concentrated collagen hydrolysate solution was prepared by dilution in distilled water at a weight ratio of 1:1. Previously, 1.5% (*w*/*v*) chitosan solution was obtained by dissolving of chitosan in 65% (*v*/*v*) aqueous acetic acid under magnetic stirring at 1000 rpm, and 60 °C for 4 h until a clear solution was obtained. HC10CC-chitosan solution with 13.6% (*w*/*v*) HC10CC was prepared by mixing HC10CC and chitosan solutions. RCG-acetic acid solution was prepared by mixing RCG with 40% (*v*/*v)* acetic acid solution, as the amount of RCG reached 25% (*w*/*v*). KH was gently mixed with 10% (*w/v*) aqueous PEO solution, as the amount of KH reached 22% (*w*/*v*). All prepared solutions were sonicated for 30 min to obtain homogeneous solutions.

Protein-based nanofibers were fabricated using a commercially TL-Pro-BM Electrospinning equipment (Tong Li Tech Co., Ltd., Bao An, Shenzhen, China), which consists of a syringe pump, a high-voltage power supplier, and a grounded conductive drum collector. Each protein/polymer/solvent solution was loaded into a Teflon syringe (10 mL volume) and fed through the tubing with a metal 21 gauge needle attached at the end. The electrospun HC10CC-, RCG-, and KH-based nanofibers were collected on a drum covered with aluminum foil. The optimal parameters for electrospinning process are summarized in Table 1. All experiments were performed at a temperature of 22.2 ± 1.1 °C and a relative humidity of 36%.

### 2.4. Characterization of HC10, HC10CC, RCG and KH Extracts

Electrical conductivity was measured with the C1010 Conductometer (Consort, Bruxelles, Belgium), with 1–100 mS/cm scale, according to EN 27888, and the viscosity was determined using a DV2T™ Viscometer (Brookfield, WI, USA). Polydispersity and zeta potential characteristics were investigated using a Zetasizer Nano-ZS device (Malvern Hills, UK). Total nitrogen and protein substances, dry substances, and total ash were measured according to ISO 5397, EN ISO 4684, and EN ISO 4047, respectively. Aminic nitrogen and average molecular weight (M_w_) were evaluated by validated in-house methods [36]. Gel permeation chromatography (GPC) was accomplished using an Agilent Technologies instrument (1260 model) (Santa Clara, CA, USA) equipped with PL aquagel-OH MIXED-H column (7.5 × 300 mm, 8 μm) and multidetection unit (260 GPC/SEC MDS containing RID, LS, and VS detectors). GPC conditions were set up at optimum values, i.e., flow rate of mobile phase containing 0.1 M Na_2_NO_3_ and 10% MeOH of 1 mL min^−1^, injection volume of the sample of 100 μL, and temperature of 35 °C for detectors and column. The GPC system was calibrated using polyethylene glycol standards with molecular weights in the range of 1000 to 16,000 g/mol.

Calculations of the M_w_ and number average molecular weight (M_n_) were performed with the Agilent GPC/SEC Software (Version 1.1, Agilent Technologies, Santa Clara, CA, USA). Circular dichroism spectra were recorded on a Jasco J-815 Spectrometer (Cremella, Italy), and the results were processed with Dichroweb software. HC10CC, RCG, and KH extracts were cast and the films obtained were analyzed by ATR-FTIR in comparison with the electrospun protein-based nanofibers.

All measurements were carried out at room temperature (25 ± 2 °C) in triplicate for each sample.

### 2.5. Characterization of Electrospun Protein-Based Nanofibers

#### 2.5.1. Morphology and Structure

The morphology and diameter of type of each electrospun protein-based nanofiber were observed by scanning electron microscopy coupled with energy-dispersive X-ray spectroscopy (SEM-EDS), using a Quanta 450 FEG scanning electron microscope (FEI, Eindhoven, The Netherlands) equipped with a field emission gun at a 1.2 nm resolution. The specimens were gold-sputtered prior to imaging, in order to increase their electrical conductivity. The diameters of minimum 50 nanofibers were measured and the results were processed using Origin 2016 software (OriginLab, Hampton, MA, USA).

Attenuated total reflectance–Fourier transform infrared spectroscopy (ATR-FTIR) analysis was done using an INTERSPEC 200-X Spectrophotometer (Tartumaa, Estonia), in transmittance mode. All spectra carried out both on the prepared films and electrospun nanofibers represent the average of 3 scans recorded at 2 cm^−1^ resolution in a 4000 to 750 cm^−1^ range, using air as background.

#### 2.5.2. Differential Scanning Calorimetry (DSC)

DSC measurements of obtained protein-based nanofibers were determined using a Mettler Toledo (Model DSC 823^e^, Calorimeter (Greifensee, Zürich, Switzerland). Samples were heated from ambient temperature up to 250 °C at a heating rate of 10 °C/min. DSC curves were processed with the aid of STAR^e^ 9.10 software from Mettler-Toledo (Greifensee, Switzerland).

#### 2.5.3. Viscoelastic Properties

The viscoelastic properties of specimens were measured by dynamic nanoindentation with a Nanoindenter XP (Tencor, former Keysight), using the dynamic testing Continuous Stiffness Measurement (CSM) module. For small-scale samples this technique can be used to locally attain the same information as dynamic mechanical analysis (DMA) in the case of bulk samples [37,38]. The measurements performed have used a special CSM-based technique that returns the complex modulus of the material as a function of frequency and separates the dynamic response of the specimen into its elastic parts (Storage modulus, *E’*) and damping or viscous component (Loss modulus, *Eʺ*) [39,40]. The indenter was oscillated at a number of 5 distinct frequencies, from 1 Hz to 45 Hz. Approximately 20 indentations were performed on each sample and the results were averaged, after excluding the outliers. The measurements yielded the complex modulus as a function of frequency. The loss factor (or tangent delta, tan δ), a key parameter used to describe the energy dissipation capability of the material was then computed as the ratio *Eʺ/E’.*

#### 2.5.4. Biocompatibility Tests

L929 mouse fibroblasts (European Collection of Authenticated Cell Cultures—ECACC) were chosen as a cell model to assess the cytotoxicity of the electrospun HC10CC, KH, and RCG nanofibers. Cells were grown in Minimum Essential Medium (MEM; Sigma-Aldrich, Steinheim, Germany) supplemented with 10% fetal bovine serum (FBS; Biochrom, Berlin, Germany) and 1% antibiotics (penicillin, streptomycin, and neomycin—Sigma-Aldrich, Steinheim, Germany) at 37 °C in a humidified atmosphere with 5% CO_2_. Cytotoxicity of the samples was evaluated by the indirect contact method according to ISO 10993-5 standard. Each of the electrospun nanofibers was immerged in MEM supplemented with 10% FBS at 37 °C for 24 h to obtain the extraction medium. Fresh culture medium was used as negative control. L929 cells were seeded (5 × 10^4^ cells/mL) in 96-well tissue culture plates and, after 24 h, the culture medium was replaced with varying concentrations of the extraction medium (0.1, 0.25, 0.5, 1, 2.5, and 5 mg/mL). Cells were maintained in standard conditions for 24 and 72 h when quantitative (MTT and LDH assays) and qualitative (cell morphology) analyses were performed.

To evaluate the cell viability, cells were incubated with 0.25 mg/mL 3-(4,5-dimethyl thiazol-2-yl)-2,5-diphenyltetrazolium bromide (MTT) solution for 3 h at 37 °C, as previously described by Craciunescu et al. [41]. The insoluble formazan crystals were dissolved with isopropanol and, after 15 min of gentle stirring at room temperature, the absorbance was recorded at 570 nm using microplate reader (Mithras LB 940, Berthold Technologies, Bad Wildbad, Germany). The concentration of the converted dye was directly correlated to the number of metabolically active viable cells. The results were calculated as percentage of viability compared to the control sample (untreated cells) considered 100% viable.

The cytotoxicity of the electrospun nanofibers was evaluated by measuring the amount of lactate dehydrogenase (LDH) released into the culture medium when cells are damaged or under stress [42]. At 24 and 72 h post-seeding, 50 μL of culture media was used to perform the LDH test using CytoTox96 kit (Promega, Madison, WI, USA), according to the manufacturer’s instructions. The LDH released into the culture medium was spectrophotometrically recorded at 490 nm using a 96-well plate reader (Tecan Sunrise, Grodig, Austria), the amount of color formed being proportional to the number of lysed cells.

Giemsa staining was used to assess cell morphology after 72 h of cell incubation in the presence of the samples. Images of the L929 cells were acquired using an Axio Observer D1 inverted microscope and AxioVision 4.6 software (Carl Zeiss, Oberkochen, Germany).

### 2.6. Statistical Analysis

All values were expressed as a mean value ± standard deviation (SD) of three independent samples (n = 3). Statistical analysis of the biocompatibility data was performed using analysis of variance (ANOVA) (95% significant level) on each pair of interest and differences at *p* < 0.05 were considered statistically significant.

## 3. Results

### 3.1. Characterization of HC10, HC10CC, RCG and KH Extracts

The main physical-chemical characteristics of collagen hydrolysate (HC10) before and after concentration (HC10CC), and RCG and KH extracts are shown in Table 2.

Collagen hydrolysate (HC10) is an accessible and inexpensive resource of collagen, which was not used in typical electrospinning processes due to its low molecular weight (3.95 kDa) and low viscosity (1.5 cP) in water (Table 2). Concentrated collagen hydrolysate (HC10CC) has associative properties, i.e., its molecular weight increased by 4.5 times as compared to HC10 [36], and its viscosity increased to 615 cP (Table 2), which makes it an interesting material for electrospinning. The conductivity of KH shows the highest value due to the high content in salts, which influences the particle size and stability. The polydispersity and viscosity of KH with similar concentration as HC10 are higher as compared to these of HC10. Aminic nitrogen and M_w_ were determined according to the validated in-house methods and compared with the results obtained by GPC in Table 3.

The values obtained for the M_w_ protein extract by GPC are in good agreement with those determined using the aminic nitrogen method (Table 2). The PDI values show the high dispersity of molecular weights of protein extracts, due to their associative properties [36].

In order to estimate if the extraction process influences the structure of proteins, the circular dichroism (CD) spectroscopy was performed (Table 4).

It was found that after the extraction processes, the collagen and keratin molecules were not totally denatured; α-helix, β-strand (the unity of β-sheets), and β-turns were identified as the most important secondary structures [43]. It is possible that after the extraction processes conducted in a water bath (not direct heating), the protein chains rewound so that small triple-helical segments continue to exist. In a separate contribution, the unfolding of the triple helix structure of collagen extracted at high temperature (110 °C) under vacuum was reported by circular dichroism [31].

### 3.2. Scanning Electron Microscopy with Energy Dispersive X-ray Spectroscopy (*SEM*-*EDS*)

**Figure 1:**
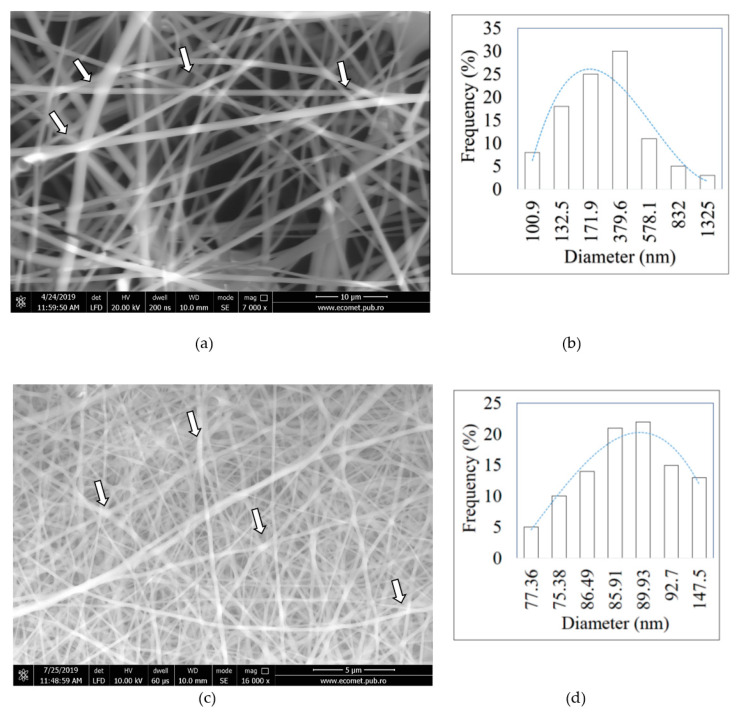

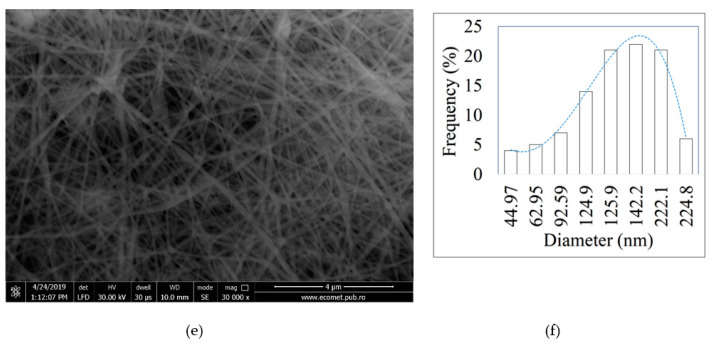
Scanning electron microscopy (SEM) images and fibers size for electrospun protein-based nanofibers: (**a**,**b**) HC10CC, (**c**,**d**) RCG, and (**e**,**f**) KH.

Figure 1a–f shows the scanning electron microscopy micrographs of the protein-based nanofibers.

Figure 1 shows that the morphology of electrospun protein-based nanofibers is influenced by their compositions and processing parameters of electrospinning. Using the chitosan solution mixed into a 65% acetic acid solution, HC10CC nanofibers with an average size of 171.9 nm were obtained (Figure 1a,b). By changing the spinning parameters, i.e., voltage of 20.53 kV, flow rate of 1.4 mL/h, and distance from needle to support collector of 8 cm, uniform RCG nanofibers without defects and an average diameter of 89.93 nm were observed (Figure 1c,d). Figure 1e,f shows the KH nanofibers with an average size of 142.2 nm and very few defects obtained by increasing the voltage up to 25 kV, while maintaining at 10 cm the distance between the needle and collector. Stripe motifs marked with white arrows can be observed for SEM images of HC10CC and RCG nanofibers, in which the ordered structure was identified by CD.

**Figure 2:**
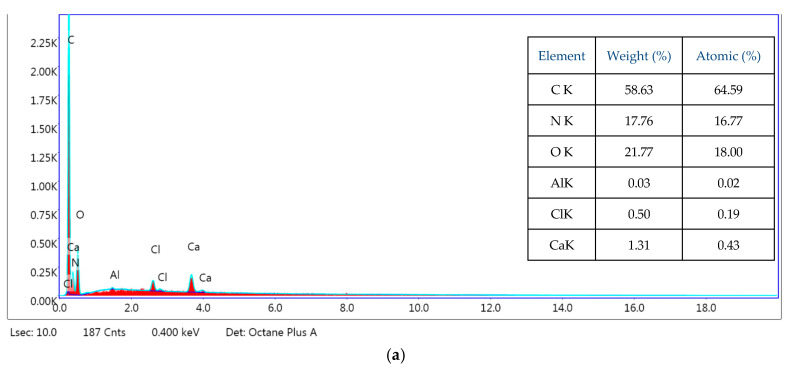

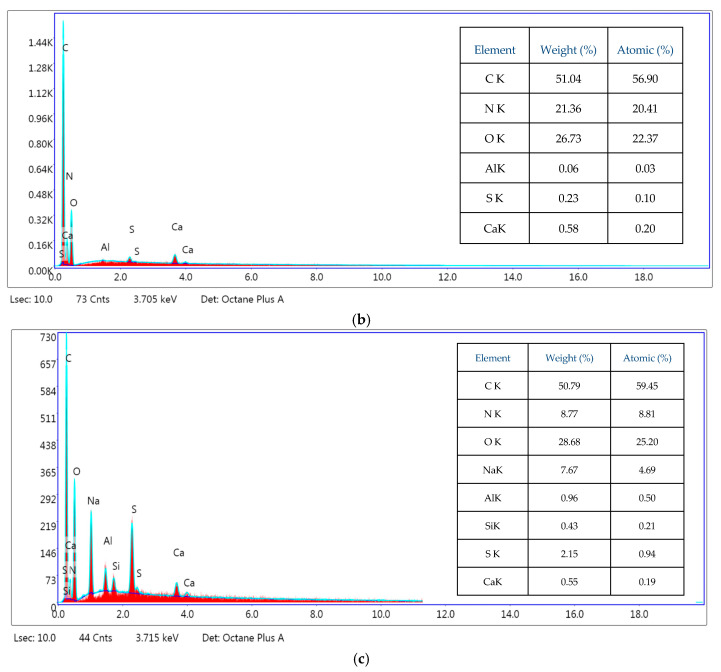
The energy dispersive X-ray patterns of nanofibers: (**a**) HC10CC, (**b**) RCG, and (**c**) KH.

The energy-dispersive X-ray (EDS) patterns of nanofibers obtained by electrospinning and their elemental composition are shown in Figure 2a–c.

The energy-dispersive X-ray (EDS) patterns of electrospun proteins nanofibers exhibit elements of the peptide functional groups of the collagen (NH, C=O), such as carbon, oxygen, and nitrogen in the ranges 50.79 to 58.63 wt.%, 21.77 to 28.68 wt.%, and 8.77 to 21.36 wt.%, respectively. The nitrogen peak increased in the case of RCG nanofibers. According to the EDS analysis of electrospun RCG and KH nanofibers, they contain a small amount of sulfur (0.23 wt.% and 2.15 wt.%, respectively). Keratin contains cysteine amino acids that form inter- and intramolecular disulfide bonds, responsible for a high stability of this protein [23]. Minor elements like aluminum, calcium, chlorine, silicon, and natrium observed in the EDS patterns of Figure 2 are due to the sample preparation of contamination during handling.

### 3.3. Attenuated Total Reflection Fourier Transform Infrared (*ATR-FTIR*) Analysis

**Figure 3:**
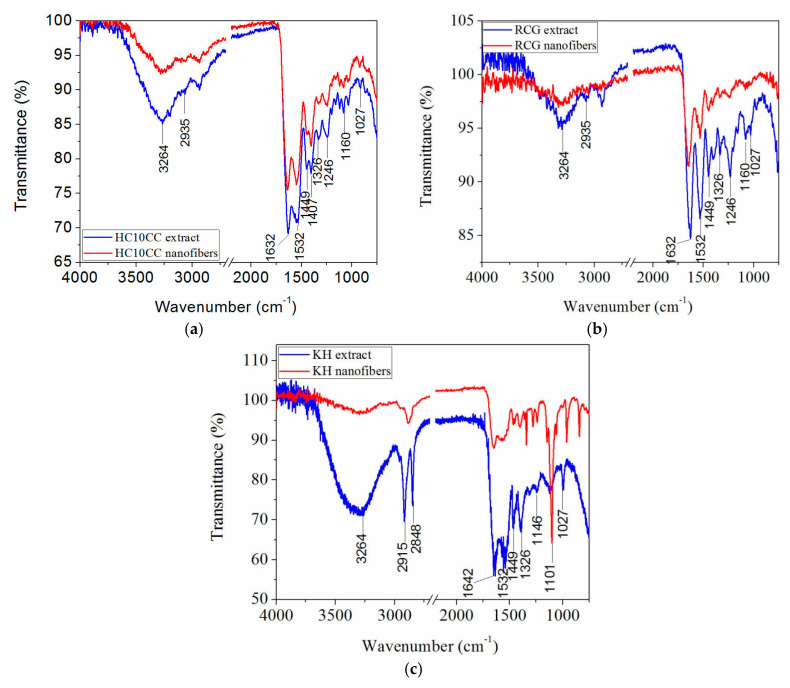
Comparative attenuated total reflectance Fourier transform infrared spectroscopy (ATR-FTIR) spectra of (**a**) HC10CC extract film and electrospun HC10CC nanofibers, (**b**) RCG extract film and electrospun RCG nanofibers, (**c**) KH extract film and electrospun KH nanofibers. The wavenumber region of interest is 4000 to 750 cm^−1^.

The purpose of ATR-FTIR spectroscopy is to demonstrate that the partial triple helix structure of protein films is preserved both for protein extracts and electrospun protein-based nanofibers. Figure 3a–c illustrates the FT-IR spectra of the prepared electrospun protein nanofibers compared with the spectra of protein extract films.

The assignment for individual peaks of HC10CC extract film indicates the presence of the prominent groups around 3264 cm^−1^ and 2935 cm^−1^ assigned to N–H stretch coupled with hydrogen bond (Amide A) and asymmetrical stretching of –CH_2_– (Amide B), respectively, see Figure 3a [44,45], also observed for RCG and KH extract films spectra in a similar wavenumbers range. The bands at 1632 cm^−1^, 1532 cm^−1^, and 1246 cm^−1^ corresponding to the C=O stretch (hydrogen bond coupled with CN stretch (Amide I)), N–H bend coupled with C–N stretch (Amide II) and C–H stretch (Amide III), respectively [27,44,45], are observed at the spectrum of HC10CC extract film. In addition, the typical peaks around 1449 cm^−1^, 1407 cm^−1^, 1326 cm^−1^, 1246 cm^−1^, 1160 cm^−1^, and 1027 cm^−1^ were observed at all proteins before and after electrospinning, which were assigned to the –CH_2_– bend, COO– symmetrical stretch, CH_2_ wagging vibration, COOH group, and C=O stretch [46]. The spectrum of the KH extract film indicates the presence of prominent groups around 2848 cm^−1^, corresponding to the stretching of the CH_2_ group arising from the addition of PEO [47] and 2915 cm^−1^ (–CH_2_–) (Figure 3c). In the case of electrospun KH nanofibers, these peaks were merged and shifted at 2874 cm^−1^. The absorption band of KH localized at 1642 cm^−1^ corresponding to α helix structure [48] was moved to 1647 cm^−1^ in the case of electrospun KH nanofibers spectrum. Furthermore, from Figure 3c it was observed that this band increased in intensity, due to the weak hydrogen bonds. The band at 1146 cm^−1^ increased in the electrospun KH nanofibers spectrum due to the planar conformation [32]. In addition, electrospun KH nanofibers indicated an intense band at 1101 cm^−1^ attributed to the cysteic acid group formed during oxidation of the cysteine thiol group (SH) in air during electrospinning process [49]. The efficiency of proteins extraction is confirmed by the ratio of about 1 between Amide III to the absorption band of –CH_2_– (1454 cm^−1^) [44], and the wavenumbers difference between Amides I and Amide II *<* 100 cm^−1^ [47] (Table 5).

All the protein extracts films and the electrospun HC10CC and KH nanofibers showed the ratio between Amide III to the absorption band of –CH_2_– very close to 1, and the wavenumber difference between Amide I and Amide II below 100 (Table 5), which means that the partial helical structure of collagen is preserved. The lowest concentration of α-helix, β-strand, and β-turns in the RCG sample measured by circular dichroism (Table 4) is confirmed by the highest value for νAmides I—νAmides II determined by ATR-FTIR. The electrospinning process slightly decreased the concentration of helical structured proteins.

### 3.4. Differential Scanning Calorimetry (DSC)

**Figure 4:**
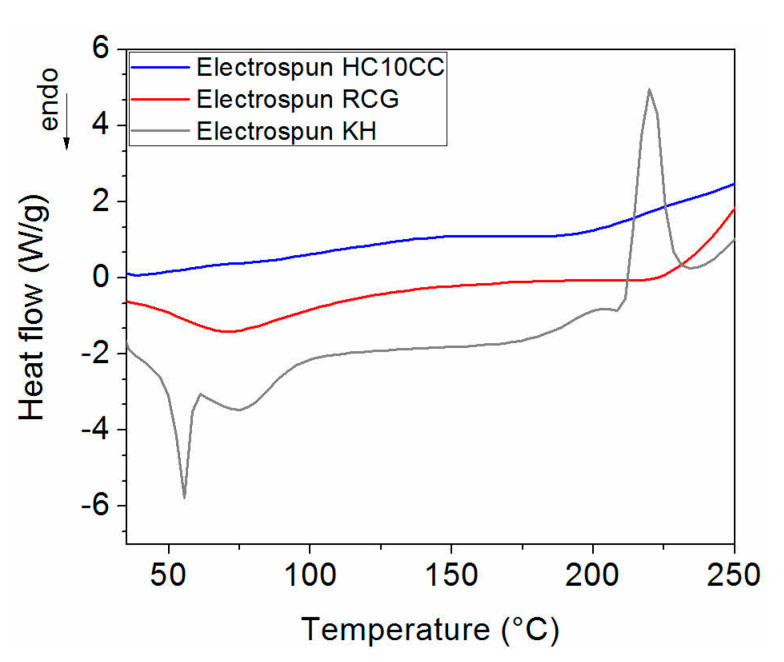
Differential scanning calorimetry curves of electrospun HC10CC nanofibers, electrospun RCG nanofibers, and electrospun KH nanofibers.

Figure 4 shows the thermal properties of electrospun protein-based nanofibers.

The denaturation temperatures for HC10CC, RCG, and KH nanofibers were observed at temperatures of 76.1, 71.6, and 76.7 °C, respectively. In the case of KH nanofibers, the peak localized at 55.6 °C is attributed to the fusion of PEO crystalline phase [32]. As the temperature increased, a degradation of the electrospun nanofibers was observed. HC10CC nanofibers exhibited the highest degradation temperature (246 °C), followed by RCG nanofibers (227 °C) and KH nanofibers (189 °C). A similar study has reported in the case keratin/PEO, which showed a degradation temperature in the range of 150 to 280 °C [48].

### 3.5. Dynamic Nanoindentation Analysis

**Figure 5:**
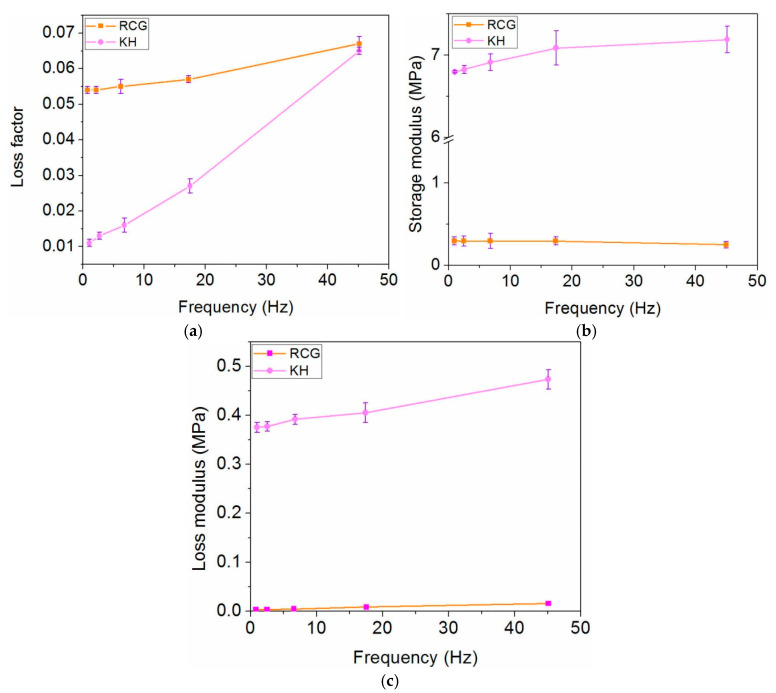
Dynamic nanoindentation analysis for electrospun RCG and KH nanofibers. (**a**) Loss factor. (**b**) Storage modulus (E′). (**c**) Loss modulus (E′′).

The dynamic nanoindentation method is a novel approach carried out to understand the mechanical properties of nanofibers, which is analogous to the dynamic mechanical analysis (DMA) test. Figure 5a–c shows the resulted viscoelastic properties versus frequency for electrospun RCG and KH nanofibers.

Viscoelastic properties of electrospun HC10CC nanofibers could not be measured due to the difficulty to prepare proper specimens. From Figure 5a–c, it is obvious that the viscoelastic properties of the electrospun RCG and KH nanofibers are different, both from the point of view of moduli magnitudes and the frequency dependence behavior.

### 3.6. In Vitro Evaluation of Nanofibers’ Biocompatibility

**Figure 6:**
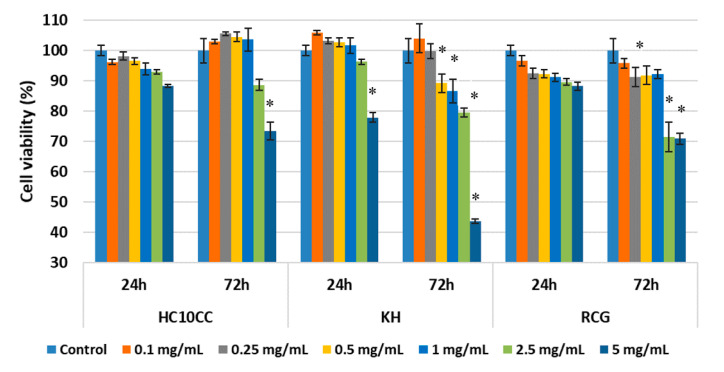
Viability of L929 mouse fibroblasts cultivated in the presence of electrospun HC10CC, KH, and RCG nanofibers for 24 and 72 h, evaluated by the MTT assay. Samples were reported to untreated cells (control) considered to have 100% viability. Data were expressed as mean values ± SD (n = 3). * *p* < 0.05, compared to the control.

Another objective of this study was to evaluate the biocompatibility of HC10CC, KH, and RCG nanofibers using the MTT assay, which evaluates the activity of mitochondrial dehydrogenases, and the LDH assay, which investigates cell membrane integrity by quantifying the LDH enzyme released in the culture medium upon cell lysis. The MTT results showed that all tested protein-based nanofibers had no cell cytotoxic activity on a wide range of concentrations. The percentages of cell viability were higher than 80% (non-cytotoxic effect) within the concentration range of 0.1 to 1 mg/mL for all the tested samples at both exposure times (24 and 72 h) (Figure 6).

HC10CC nanofibers were also cytocompatible at the concentration of 2.5 mg/mL, whereas a moderate cytotoxicity was recorded at the concentration of 5 mg/mL after 72 h (cell viability of 73.43%). Cell viability was maintained above 80% for KH nanofibers at the concentration of 2.5 mg/mL, but significantly decreased below 50% at 72 h post-seeding (43.72%, *p* < 0.05). Similar cell viability values were obtained after 24 h for RCG sample at the concentrations of 2.5 and 5 mg/mL (89.65 and 88.28%, respectively), whereas a moderate cytotoxic effect was observed after 72 h (71.58% at the concentration of 2.5 mg/mL and 70.91% at the concentration of 5 mg/mL).

**Figure 7:**
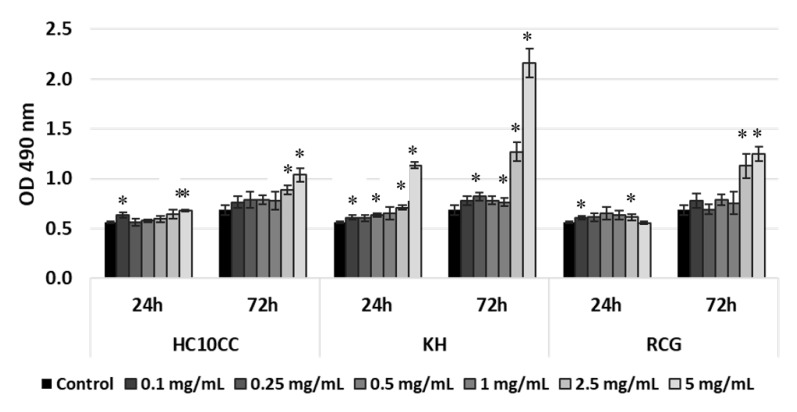
Evaluation of LDH activity released into the culture medium by the L929 cells grown in the presence of HC10CC, KH, and RCG electrospun nanofibers at 24 and 72h post-seeding. Data are expressed as mean value ± SD (n = 3). * *p* < 0.05, compared to the untreated cells (control).

In addition, to determine the degree of cell death as a consequence of the cytotoxicity displayed by the tested electrospun nanofibers, secreted LDH levels were measured after 24 and 72h of cell treatment. Thus, the L929 cells seeded in the presence of the three electrospun nanofibers showed low LDH released in the culture medium at 24 h post-seeding within the entire concentration range tested (0.1–5 mg/mL), except for KH sample at a concentration of 5 mg/mL, and also after 72 h of culture, within the concentration range of 0.1 to 1 mg/mL (Figure 7).

These results suggest that none of these nanofibers exhibited cytotoxic effects at the above mentioned concentrations. However, higher levels of LDH activity were recorded after 72 h for HC10CC at the concentration of 5 mg/mL and for KH and RCG nanofibers at the concentrations ranging between 2.5 and 5 mg/mL (Figure 7). These results were well correlated with those obtained by the MTT assay, suggesting cytotoxic activity starting from the concentration of 2.5 mg/mL, particularly for KH and RCG nanofibers.

**Figure 8:**
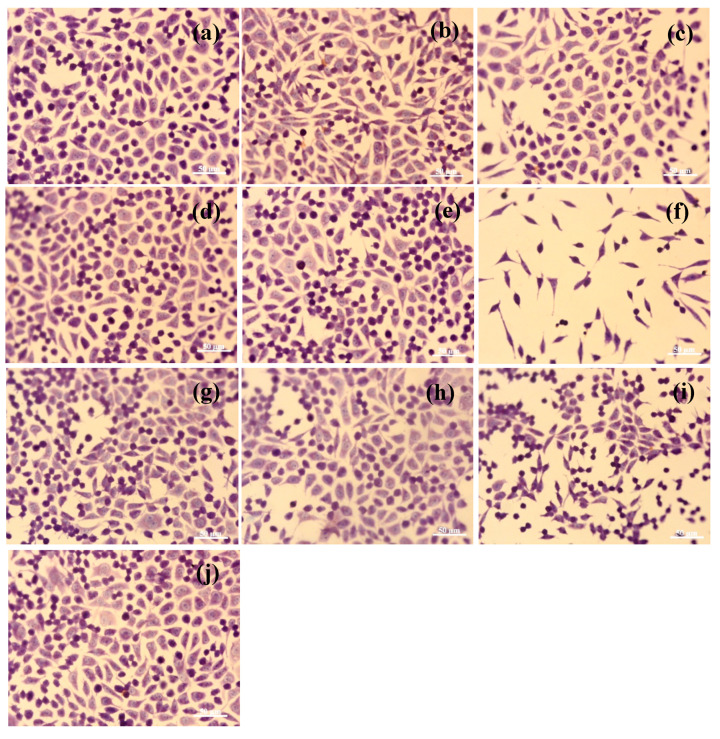
Light microscope images of L929 fibroblast cells treated with electrospun HC10CC nanofibers at concentrations of 0.1, 1, and 5 mg/mL (**a**–**c**); electrospun KH nanofibers at concentrations of 0.1, 1, and 5 mg/mL (**d**–**f**); electrospun RCG nanofibers at concentrations of 0.1, 1, and 5 mg/mL (**g**–**i**); and untreated cells (control—**j**) for 72 h (Giemsa staining). Scale bar = 50 μm.

The quantitative results of cell cytotoxicity were confirmed by cell morphological analysis after Giemsa staining. Light microscope images revealed that L929 fibroblast cells exposed to different electrospun nanofibers maintained their normal morphological appearance, similar to that of the control (Figure 8j), up to a concentration of 1 mg/mL (Figure 8a,b,d,e,g,h). Cells exhibited a fibroblast-like phenotype, with euchromatic nuclei and 1–4 nucleoli, clear cytoplasm, and cytoplasmic extensions. Cells also presented a density similar to that of the control, covering around 80–85% of the well surface. At the highest tested concentration of 5 mg/mL, cell density decreased significantly, particularly for KH sample (Figure 8f), and obvious morphological changes in cell shape and cytoplasm and nuclei appearance were also observed.

## 4. Discussion

The electrospinning process represents a sustainable nanotechnology addressing large-scale material production. In this paper, nanofibers of indigenous natural resources based on collagen from bovine hides, rabbit skin glue, and sheep wool keratin originated from valuable by-products from fur and leather industry were successfully obtained by electrospinning. In order to set the optimal parameters for achieving the electrospun protein-based nanofibers, different polymers and solvent concentrations, amount of protein, flow rate, distance from the needle to collector, and voltage were tested. Concentrated collagen hydrolysate (HC10CC) shows a higher viscosity as compared to diluted collagen dispersion and associative properties, which make this material suitable for electrospinning in combination with the chitosan solution. Good spinnable properties of collagen hydrolysate are obtained by the increased viscosity with its concentration [50]. Electrical conductivity and ionic properties of HC10CC nanofibers are substantially diminished due to the peptide molecule associations. First, HC10CC nanofibers were dissolved in distilled water and tested in the following conditions; voltage of 22.32 kV, flow rate of 2.6 mL/h, and distance between the needle to aluminium collector of 7 cm. The electrospraying effect was frequently observed. Then, the electrospinning process was conducted by introduction of 1.5% (*w*/*v*) chitosan dissolved in 65% aqueous acetic acid, as the amount of HC10CC reached 13.6% (*w*/*v*). In this case, the voltage was decreased to 18.81 kV. Chitosan is a unique cationic polysaccharide, an abundant and renewable natural polymer that shows many intrinsic properties, such as antioxidant, lipid-lowering and antimicrobial activities, film-forming and gelling properties, encapsulation potential, biodegradability, biocompatibility, non-toxicity, antitumor, antimicrobial, and adsorption activity [51,52,53]. Electrostatic interactions occurred between the functional groups of collagen and chitosan, with the forming of polyanion–polycation complex and new hydrogen bonding networks [54,55], which can provide complementary performance and synergy [56]. The spinability of the collagen–chitosan solution is improved due to the miscibility of components that is assigned to the intermolecular interactions created between functional groups. This miscibility is further responsible for the mechanical properties of new materials with potential for biomedical applications. Similar, collagen–chitosan complex was prepared in the mixture of 1,1,1,3,3,3 hexafluoro-2-propanol (HFP)/trifluoroacetic acid (TFA) and tried for electrospinning [27,54]. The authors reported that the fiber’s size decreased with increasing of the content of chitosan in the collagen–chitosan complex due to the increased charge density of the polymer solution. The high viscosity value of rabbit collagen glue (RCG) (18,670 cP), even at low concentration, seems to be the most important characteristic for the electrospinning process. To the best our knowledge, the RCG was used for the first time in our work as a raw material for collagen nanofibers processing, due to the outstanding soldering properties as compared to other mammalian collagen materials. In the case of electrospun RCG dissolved in distilled water, it was observed that the solution was prevented to flow due to the gluing property. In order to overcome this inconvenience, RCG was mixed with acetic acid solution. This solution was electrospun at a voltage of 17.89 kV, a flow rate of 0.4 mL/h, and a distance from the needle to collector of 8 cm, but frequently beads were observed. By changing the voltage to 20.53 kV, the flow rate to 1.4 mL/h, and the distance to collector to 8 cm, the adequate nanofibers were collected. Other authors also used a diluted acetic acid solution for the preparation of nanofibers based on biopolymers with structural similarity to ECM, due to the reducing risk of toxic residuals found in the nanofibers [57]. KH dissolved in distilled water was electrospun by applying a voltage of 11.09 kV and a flow rate in the range of 1.1 to 0.6 mL/h. In this situation, the solution was prevented to elongate as a continuous jet. Harmful solvents, such as 2,2,2-trifluoroethanol, formic acid, chloroform/N-dimethylformamide, and 1,1,1,3,3,3-hexafluoro-2-isopropanol, were used to dissolve keratin for the electrospinning process [58]. These solvents are toxic for biomedical applications and break the disulfide bridges of keratin, therefore losing its rigidity. PEO was selected as a good candidate for improving the quality of nanofiber deposition [32]. By adding PEO and increasing the voltage up to 25 kV, the keratin nanofibers were collected.

The M_w_s of protein extracts evaluated and validated by in-house methods are in good agreement with the GPC determinations. The structure of protein extracts evaluated by circular dichroism spectroscopy showed that the secondary structure of proteins was partially preserved after extraction conditions. The helix structure in the case of KH after alkaline extraction was also reported by other authors [59].

The SEM observations of electrospun protein-based nanofibers revealed that the HC10CC resulted in larger nanofiber size (171.9 nm) than RCG (89.93 nm) and KH nanofibers (142.2 nm). The large nanofibers size of HC10CC could be due to the molecular weight of collagen hydrolysate of 17.8 kDa. Fibers with a diameter larger than 2 μm were obtained in the case of drug-loaded collagen/PLA [29], due to the structure of collagen fibers, which usually form nanofibers with length of ~100 nm. The high ratio of surface area to volume of the electrospun RCG and KH nanofibers will be promising for medical applications where the growth and proliferation of cells culture are required. A partially preserved native structure for HC10CC and RCG nanofibers, like a stripe motif [31], could be observed from the SEM images. This observation proves that the partial secondary structure of proteins is preserved, with beneficial effects on the nanofibers biocompatibility. The preservation of triple helix structure was also confirmed for all the protein extracts and electrospun HC10CC and KH nanofibers by the evaluating the ratio between Amide III to the absorption band of -CH_2_- with value of about 1. The spectra of protein extract films revealed the wavenumber difference between Amides I and II < 100 cm^-1^ compared with those of electrospun protein nanofibers. This spectral behavior suggests that the protein chains were reorganized during electrospinning process, as ß-sheet and ɑ-helix conformation secondary structures. Electrospun RCG nanofibers showed a wavenumber difference ˃100, which indicates the presence of denatured collagen. The partial helix identification in electrospun protein-based nanofiber structures as compared to the protein extract films is a new discovery of this research with great potential for advanced biocompatible material design as compared to similar known products based on biopolymers. The triple helix structure of electrospun HC10CC nanofibers has been confirmed by other authors [8,60], although no evidence of triple helix has been reported to date [49].

DSC analysis reveals that the electrospun HC10CC, RCG, and KH nanofibers showed an improvement in their thermal stability, as compared with the denaturation temperature of native hydrated collagen (65 °C) [61]. This favorable behavior could be attributed to the effect of chitosan and PEO polymers used for the electrospinning of collagen and keratin extracts, which is in agreement with the C–H stretch (Amide III), proving the stability of triple helix [48]. During the thermal analysis, the electrospun protein-based nanofibers did not show water evaporation; it is possible that this process overlapped with the denaturation of proteins, maybe due to the high ratio of surface area to volume of the nanofibers, as shown in SEM observation.

The E′′ and loss factor of the electrospun RCG nanofibers show a continuous increase over the range of frequency used (about 6× in all), while the storage modulus stays constant up to 20 Hz, showing a slight decrease at 45 Hz. All measured mechanical parameters for electrospun KH nanofibers are fairly constant over the frequency range; both storage and loss moduli are several tens of times larger than for electrospun RCG nanofibers, while the loss factor is 5 to 10× larger over on the tested frequency range. Regarding the energy dissipation capacity, this material proves superior capabilities compared to RCG nanofibers. The results can be explained by the smaller diameter size of RCG nanofibers compared to KH nanofibers. It is possible that the hard substrate of electrospun KH nanofibers influences the deformation of nanofibers.

These results, which combine both in vitro quantitative and qualitative methods, show good cytocompatibility of the electrospun KH and RCG nanofibers at concentrations ranging between 0.1 and 1 mg/mL, whereas HC10CC nanofibers exhibit no cytotoxic activity up to a concentration of 2.5 mg/mL. Our observation is consistent with previous studies, which reported biocompatible electrospun keratin and collagen/chitosan matrices that are suitable for tissue engineering and wound healing [62,63,64]. Thus, Xing et al. [63] developed keratin-PEO nanofibers at a weight ratio of 90:10, which also supported the adhesion and proliferation of NIH 3T3 fibroblasts. Using human hair keratin, Sow et al. [64] managed to electrospun keratin-PEO blends in a weight ratio of 60:1; the matrices have shown no cytotoxicity while providing a structural environment for cell attachment and growth. Cell proliferation was also promoted when keratin blends were mixed with various synthetic polymers, such as poly(lactic acid) or poly(hydroxybutyrate-co-hydroxyvalerate) [65,66]. Electrospun collagen/chitosan nanofibrous membranes showing good in vitro biocompatibility, which also induced cell migration and proliferation, were also fabricated in order to be used as potential biomaterials for skin or bone regeneration [62,67,68]. Future work will be devoted to the modification of the electrospun protein-based nanofibers surface by inclusion of functional bioactive compounds for tissue engineering applications.

## 5. Conclusions

Different proteins from bioactive by-product resources were extracted and characterized in order to obtain electrospun nanofibers. The experiments showed that the concentrated collagen hydrolysate extracted from bovine leather by-products, rabbit skin collagen glue, and keratin hydrolysate from wool waste have the properties required for electrospinning processing. The concentrated collagen hydrolysate (HC10CC)–chitosan, RCG–acetic acid, and KH–poly(ethylene oxide) (PEO) solutions were straightforwardly processed by electrospinning. Accordingly to the circular dichroism spectroscopy performed on the extracted protein, it was found that the extraction process did not totally denature collagen structure. Preservation of the helical structure of collagen was also assessed by measuring the ratio between Amide III to the absorption band of –CH_2_– and the wavenumbers difference between Amides I and Amides II. A partially preserved native structure of HC10CC and KH nanofibers was confirmed by SEM analysis, while the DSC analysis showed an increase in the denaturation temperature as compared with native collagen. KH nanofibers showed superior dynamic mechanical properties as compared to RCG nanofibers. Finally, in vitro experiments demonstrated the cytocompatibility of all electrospun nanofibers, with HC10CC presenting the most promising result, which recommends the possible use of keratin nanofibers for the bone tissue engineering, while the collagen and rabbit collagen glue nanofibers can be used as biomimetic extracellular matrix for the regeneration of tissues.

## Figures and Tables

**Table 1:** Optimal parameters for obtaining of electrospun HC10CC-, RCG-, and KH-based nanofibers.

Nanofibers	Flow Rate (mL/h)	Voltage (kV)	Distance from Needle to Collector (cm)
HC10CC/Chitosan	3.0	18.81	12
RCG/Acetic acid	1.4	20.53	8
KH/PEO	1.0	25.00	10

**Table 2:** Physical-chemical characteristics of collagen hydrolysate, concentrated collagen hydrolysate, rabbit collagen glue, and keratin hydrolysate extracts.

Characteristics	Unit	HC10	HC10CC	RCG	KH
Dry substance	%	9.07	57.25	11.78	9.02
Total ash *	%	7.72	6.57	1.61	13.73
Total nitrogen *	%	14.99	14.62	17.32	14.40
Protein substance *	%	84.24	82.16	97.28	80.84
Aminic nitrogen **	%	1.60	0.83	0.87	1.03
M_w_	Da	3,950	17,800	15,000	12,000
pH	pH units	8.14	8.91	7.50	11.84
Electrical conductivity	μS/cm	8,400	2,360	820	13,700
Viscosity	cP	1.5	615	18,670	47
Average particle size	nm	640.2	807	858.2	1,822
Polydispersity		0.79	0.76	0.79	0.91
Zeta potential	mV	−7.28	−3.89	−12.2	−11.4

* values reported at dry substance; ** value reported at protein content.

**Table 3:** M_w_, M_n_, and polydispersity index (PDI) of HC10CC, RCG, and KH extracts used for preparation of electrospun protein-based nanofibers.

Sample	M_w_ (g/mol)	M_n_ (g/mol)	PDI
HC10CC	17,124	16,075	1.06
RCG	15,479	13,509	1.15
KH	13,344	7647	1.74

**Table 5:** Ratio between Amide III to the absorption band of –CH_2_– (~1454 cm^−1^) and ν_Amides I_ - ν_Amides II_ evaluated from ATR-FTIR spectra for extracted proteins compared with electrospun samples.

Sample	Amide III/(~1454 cm^−1^)	νAmides I—νAmides II (cm^−1^)
HC10CC extract film	1.06	75.5
Electrospun HC10CC	1.05	87.0
RCG extract film	1.02	93.4
Electrospun RCG	1.01	109.5
KH extract film	0.99	88.5
Electrospun KH	1.01	93.7

**Table 4:** The structure of extracted proteins measured by circular dichroism (CD).

Sample	α-Helix (%)	β-Strand (%)	β-Turns (%)	Random Coil (%)
HC10CC	17	33	15	35
RCG	5	24	14	57
KH	6	34	1	41
